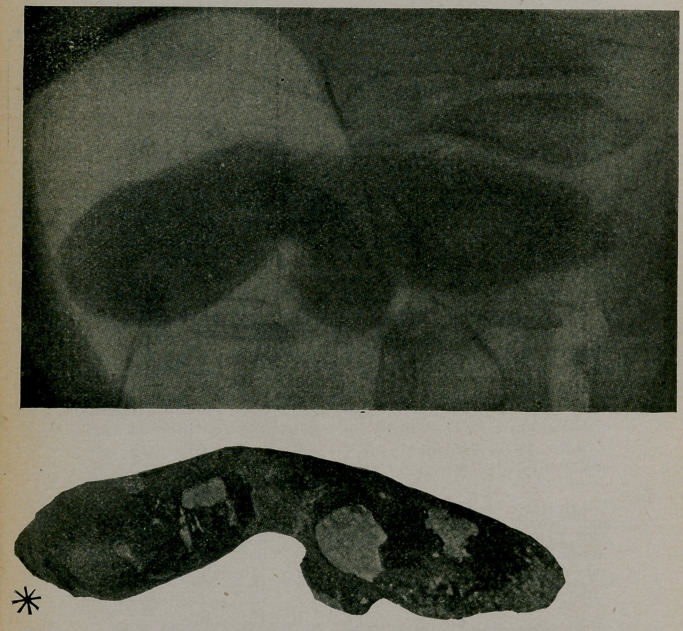# Giant Calculus of Ureter

**Published:** 1915-11

**Authors:** 


**Giant Calculus of Ureter.** P. A. Specklin, Strasburg, article translated for Am. Jour. of Urol., July, reports a case in a man aged 48 of 12 years duration. Operation revealed calculus of pelvis, kidney enlarged and cystic, also calculus of ureter, weighing 51 grams, 11c. m. in long axis, 12 c. m. measured on the curve. The pointed extremity was seen by cystoscope. Rference is made to the following large ureteral calculi: Federoff, 19 c.m., weight 52 grams; Rovsing, 18 c. m., width of bean; Israel, 13 c. m. x about 3, 54.4 grams; ibid. 17x3 c. m.; Pozzi, 34.5 grams; Lloyd, 5½ x inches with circumference of 2½ inches.

## Figures and Tables

**Figure f1:**